# REBECGA: Registro Brasileiro de Cardiopatia e Gravidez. Estudo Multicêntrico Epidemiológico sobre Cardiopatias na Gravidez: Etapa Retrospectiva

**DOI:** 10.36660/abc.20240807

**Published:** 2025-08-06

**Authors:** Walkiria Samuel Avila, Alexandre Jorge Gomes de Lucena, Claudia Maria Vilas Freire, Fabio Bruno da Silva, Ivan Romero Rivera, Juliana Rodrigues Soares Oliveira, Maria Alayde Mendonça Rivera, Regina Coeli de Carvalho, Vicente Avila, Flávio Tarasoutchi

**Affiliations:** 1 Hospital das Clínicas Faculdade de Medicina Universidade de São Paulo São Paulo SP Brasil Instituto do Coração do Hospital das Clínicas da Faculdade de Medicina da Universidade de São Paulo, São Paulo, SP – Brasil; 2 Hospital Agamenon Magalhães Recife PE Brasil Hospital Agamenon Magalhães, Recife, PE – Brasil; 3 Universidade Federal de Minas Gerais Belo Horizonte MG Brasil Universidade Federal de Minas Gerais, Belo Horizonte, MG – Brasil; 4 ECOCENTER Belo Horizonte MG Brasil ECOCENTER, Belo Horizonte, MG – Brasil; 5 Instituto Dante Pazzanese de Cardiologia São Paulo SP Brasil Instituto Dante Pazzanese de Cardiologia, São Paulo, SP – Brasil; 6 Universidade Federal de Alagoas Faculdade de Medicina Maceió AL Brasil Universidade Federal de Alagoas Faculdade de Medicina, Maceió, AL – Brasil; 7 Secretaria de Estado da Saúde do Ceará Fortaleza CE Brasil Secretaria de Estado da Saúde do Ceará (SESA), Fortaleza, CE – Brasil; 8 Hospital do Servidor Público Municipal São Paulo SP Brasil Hospital do Servidor Público Municipal, São Paulo, SP – Brasil

**Keywords:** Registro, Cardiopatias, Gravidez, Mortalidade Materna

## Abstract

**Fundamento:**

A doença cardíaca, que afeta cerca de 4% das gestações, é a principal causa não obstétrica de morte materna. Estima-se que 40% dessas mortes poderiam ser evitadas com a melhor compreensão sobre os riscos da gravidez.

**Objetivos:**

Desenvolver o Registro Brasileiro de Cardiopatias na Gravidez (REBECGA) para estudar a prevalência, as complicações e a mortalidade materna associadas às cardiopatias durante a gestação e após o parto.

**Métodos:**

Estudo multicêntrico e retrospectivo da evolução da gestação até 12 meses após seu término, em mulheres com doenças cardíacas, com análise das variáveis preditivas dos desfechos maternos. Adotou-se nível de significância bilateral de 5% para a análise estatística.

**Resultados:**

Incluiu-se 638 gestantes com os seguintes diagnósticos: doença valvar (37,8%), cardiopatias congênitas (35,7%), arritmias sem lesão cardíaca estrutural (14,7%), cardiomiopatias (11,3%) e outras cardiopatias (6,4%). As principais complicações foram: insuficiência cardíaca (16,7%), arritmias (10,7%) e síncope (8,0%), com 18 óbitos (2,8%) maternos (2,8%) sendo 10 no puerpério. Parto cesárea foi indicado em 62,9%, com 25,7% de prematuros e 3,2% de perdas fetais. A análise multivariada identificou classe de risco IV da Organização Mundial da Saúde modificada (OR 3,51; IC 95% 1,75-7,04; p<0,001); CF III/IV da New York Heart Association (NYHA) (OR 2,27; IC 95% 1,12-4,60; p=0,023) e hipertensão pulmonar (OR 2,45; IC95% 1,05-5,70; p=0,037) como variáveis preditivas de complicações e óbitos maternos.

**Conclusões:**

O REBECGA revelou a prevalência da doença valvar, a insuficiência cardíaca como principal complicação, o puerpério como um período crítico para os óbitos maternos e identificou variáveis preditivas de risco para a gravidez em mulheres com doenças cardíacas.

## Graphical abstract

**Figura Central:**
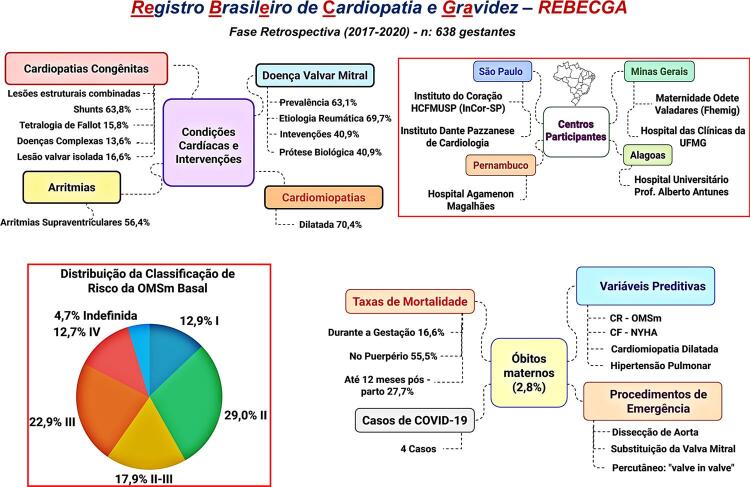
REBECGA: Registro Brasileiro de Cardiopatia e Gravidez. Estudo Multicêntrico Epidemiológico sobre Cardiopatias na Gravidez: Etapa Retrospectiva

## Introdução

As cardiopatias afetam cerca de 4% das gestações no Brasil,^1^ sendo a principal causa não obstétrica de morte materna durante a gravidez e após o parto.^2^ Esse cenário decorre do aumento da prevalência de fatores de risco cardiovascular em mulheres jovens, o adiamento da gravidez para idades mais avançadas e a maior sobrevida de mulheres com cardiopatias manifestas na infância e adolescência.

Cerca de 80% das mortes maternas por cardiopatias são evitáveis, com dois terços ocorrendo nos primeiros 12 meses pós-parto.^3^ Essas mortes são frequentemente atribuídas à falta de diagnóstico precoce e tratamento especializado, e a falhas no sistema de saúde.^4^

A maioria das recomendações sobre cardiopatia e gravidez baseiam-se em evidências de nível C, fundamentadas principalmente em opiniões de especialistas e registros multicêntricos.^5 - 11^ Nesse contexto, a classificação de risco da Organização Mundial da Saúde modificada (OMSm) tem demonstrado boa sensibilidade na predição de complicações maternas, sendo uma ferramenta valiosa tanto para o aconselhamento prévio à concepção quanto na condução das gestações de mulheres cardiopatas^5 , 12^( Tabela 1 Suplementar ).

No entanto, lacunas persistem no Brasil, onde escores internacionais são limitados devido a diferenças epidemiológicas, sociais e demográficas.^13^ Nesse cenário, a criação do Registro Brasileiro de Cardiopatia e Gravidez (REBECGA) surge como uma necessidade para fornecer uma visão realista das cardiopatias na gravidez no Brasil, alinhando o país às iniciativas globais.

O principal objetivo do REBECGA é determinar a prevalência de cardiopatias, avaliar a ocorrência de complicações cardíacas e mortalidade materna relacionadas à gravidez. Além disso, busca identificar fatores preditivos de complicações e óbitos maternos, bem como descrever a evolução obstétrica e fetal.

## Métodos

O REBECGA é um estudo observacional multicêntrico acessível a hospitais de todo o Brasil com infraestrutura adequada para acompanhar mulheres com diagnóstico prévio de doença cardíaca, desde a gestação até 12 meses após o parto, mesmo que participem de outros ensaios clínicos. O período de 12 meses pós-parto foi considerado para identificar as circunstâncias da mortalidade materna tardia, conceitualmente definida como até 12 meses após o término da gestação. O estudo é composto por duas etapas: 1) uma etapa retrospectiva, já concluída, com dados de gestantes atendidas entre 2017 e 2020 (conteúdo desta publicação); 2) uma etapa prospectiva, em andamento, com término previsto para 2027.

Os dados considerados para estudo foram: 1) diagnóstico da cardiopatia; 2) classificação de risco OMSm ( CR I, II, II-III, IV - OMSm), 3) classe funcional da *New York Heart Association* (CF I,II,III,IV-NYHA)); 4) fatores complicadores – disfunção ventricular, hipertensão pulmonar (HP) , hipoxemia, fibrilação atrial (FA), lesões residuais após intervenção cirúrgica ou percutânea, disfunção de enxertos ou de próteses); 5) eventos cardíacos anteriores à gestação; 6) histórico de cirurgia ou intervenção e 7) antecedentes obstétricos.

Os critérios de exclusão do estudo foram: pacientes sem diagnóstico definido ou em investigação de cardiopatia, HP sem envolvimento cardíaco primário e arritmias cardíacas sem registro documentado.

A análise da evolução nos trimestres da gestação, no puerpério (período até 42 dias após o parto) e até 12 meses após o término da gestação, considerou a ocorrência de: 1) complicações cardíacas – insuficiência cardíaca (IC), “nova” arritmia, tromboembolismo, hipoxemia, síncope, infarto agudo do miocárdio, dissecção de aorta, endocardite infecciosa); 2) óbitos maternos; 3) hospitalização para tratamento das complicações cardíacas; 4) necessidade de tratamento intervencionista (cirúrgico ou percutâneo) e 5) complicações obstétricas agrupadas em abortamento, hipertensão gestacional, hemorragia pós-parto e infecção. Os óbitos maternos foram analisados “caso a caso”, considerando as características clínicas prévias à gestação, causa do óbito e a sobrevida fetal.

As condutas adotadas, incluindo medidas gerais e preventivas, terapêutica farmacológica e/ou intervencionista, seguiram às recomendações convencionais estabelecidas pelas Diretrizes em Cardiopatia e Gravidez.^5 - 7^

Realizou-se uma análise descritiva das informações relacionadas aos tipos de parto, à anestesia utilizada e aos dados dos recém-nascidos, incluindo perdas fetais, prematuridade e cardiopatias congênitas, com base em informações disponíveis nos autorrelatos das pacientes ou prontuários.

Foram realizadas análises para avaliar a relação entre as doenças cardíacas, as características clínicas das pacientes e a ocorrência de complicações e óbitos maternos.

### Registro e gerenciamento de dados

Os dados foram registrados na plataforma Research Electronic Data Capture (REDCap)^14^ pelos pesquisadores de cada centro participante e monitorados pela *Academic Research Organization* (ARO) do Instituto do Coração (InCor) – FMUSP. O estudo foi observacional e descritivo, sem intervenções e sem riscos às participantes, foi aprovado pelo Comitê de Ética em Pesquisa do Hospital das Clínicas da Faculdade de Medicina da Universidade de São Paulo (HCFMUSP) sob os protocolos SDC5232/21/007 CAAE: 42739321.9.1001.0068.

### Análise estatística

A análise estatística foi realizada com o software R 4.2.2 e gráficos elaborados com o pacote ggplot2.^15^ As variáveis contínuas foram apresentadas como médias e desvios-padrão ou medianas e intervalos interquartis, dependendo da distribuição. A normalidade da distribuição foi avaliada pelo teste de Kolmogorov-Smirnov. Variáveis categóricas foram descritas por frequências absolutas e relativas. O teste de Fisher e o qui-quadrado avaliaram associações entre variáveis categóricas. ANOVA *one-way* e testes t não pareados ou de Wilcoxon-Mann-Whitney foram usados para comparar variáveis contínuas entre os grupos. Um modelo de regressão logística binária foi ajustado para identificar variáveis associadas à ocorrência de complicações e/ou óbito materno, com seleção inicial de variáveis com p < 0,10 no teste univariado e método *stepwise* . Para a avaliação do desempenho preditivo dos modelos, considerou-se a utilização de curvas ROC. Para os testes estatísticos, foi adotado um nível de significância de 5% e todos foram considerados como bicaudais.

## Resultados ( Figura Central )

No período de 2017 a 2020, dados de 638 gestantes com doenças cardíacas foram incluídos na etapa retrospectiva do Registro-REBECGA, que contou com a participação de seis centros de cardiologia ( Figura Central e Figura Suplementar 1 ). As pacientes do estudo tinham, em média, 30,4 ± 7,3 anos, com predominância de mulheres brancas e solteiras. Metade possuía ensino fundamental ou médio completo, e 62,7% relataram gestações anteriores ( Tabela Suplementar 2 ). Quanto ao aconselhamento reprodutivo prévio, apenas 32,2% planejaram a gravidez, 57,3% receberam orientação sobre anticoncepcionais e 64,9% tiveram orientação sobre os riscos da gravidez.

A análise da distribuição das doenças cardíacas ( Figura Suplementar 2 ) destacou a prevalência da doença valvar, muito próxima à das cardiopatias congênitas, e um percentual de arritmias sem lesão estrutural superior ao de cardiomiopatias. Houve predomínio da lesão valvar mitral reumática sendo que a intervenção cirúrgica ou percutânea prévia à gestação teve o implante de prótese biológica (PB) como o procedimento mais frequente ( Figura Suplementar 3A ).

Entre as cardiopatias congênitas, predominaram as lesões combinadas, as comunicações intercavitárias (“shunts”) e a tetralogia de Fallot, como cardiopatia cianogênica ( Figura Suplementar 3B ).

No grupo das cardiomiopatias, o fenótipo dilatado foi o mais frequente, distribuídos de forma semelhante entre a idiopática e periparto ( Figura Suplementar 3C ). Entre as doenças arritmogênicas isoladas, as arritmias supraventriculares predominaram, sendo que 53 (56,4%) pacientes haviam sido submetidos a intervenção percutânea antes da gestação ( Figura Suplementar 3D ).

As características clínicas das pacientes no primeiro atendimento da gestação ( Tabelas Suplementares 3 A, B, C, D, E ) mostrou que cerca de um terço (36,1%) das pacientes foram classificadas em risco III/IV-OMSm ( Figura Central ); 71/598 (11,2%) pacientes estavam em CF-III/IV-NYHA. No total, foram identificados 224 fatores complicadores (36,5%) e 187 eventos cardíacos prévios à gestação (29,2%) entre as pacientes incluídas, podendo um mesmo caso apresentar mais de um fator ou evento.

A análise longitudinal da evolução materna ( Tabela Suplementar 4 ) nos trimestres da gestação mostrou a progressão da CF-NYHA até o puerpério, com necessidade de hospitalizações, ajustes na terapêutica e indicações de intervenções cirúrgicas ou percutâneas nos casos refratários ao tratamento clínico. As complicações cardíacas mais frequentes foram IC, arritmias e síncope. Ao longo dos 12 meses seguintes à gestação, a taxa de complicações permaneceu elevada e mais da metade dos óbitos maternos aconteceu no puerpério, com um total de 10 em 18 (55,6%) ocorrências fatais. A frequência dos medicamentos prescritos na gestação e após o parto, de acordo com análise de 602 pacientes, está resumida na Tabela Suplementar 5 .

Foram registrados 18 (2,8%) óbitos maternos, três (16,6%) durante a gestação, 10 no puerpério (55,5%) e cinco (27,7%) ao longo de 12 meses após o parto descritos caso a caso. Os casos 8, 9, 10 e 14 apresentaram infecção por Covid-19 que antecedeu a morte materna, e houve um natimorto (caso 10) ( Tabela Suplementar 6 ).

Foram realizados três procedimentos em caráter de emergência: 1º ) caso 13, correção de dissecção de aorta, com implante de coronárias, na 36ª semana de gestação após a retirada do feto vivo e saudável, com evolução para óbito materno no segundo dia de pós-operatório; 2º) caso 10, reoperação de troca valvar mitral devido à calcificação de prótese, no 30º dia pós-parto com evolução para óbito no segundo dia do pós-operatório, e 3º) caso 12, realizado procedimento percutâneo de “ *valve in valve* ,” na 12ª semana de gestação, em paciente com prótese calcificada em IC aguda, evoluindo para óbito no primeiro pós-operatório.

A análise das características basais das pacientes e da frequência de complicações maternas e fetais mostrou diferenças (p < 0,05) entre os tipos de doenças cardíacas, para as variáveis idade, OMSm, CF-NYHA, fatores complicadores, histórico de eventos cardíacos e intervenções prévias à gestação ( Tabela Suplementar 7 ).

As complicações cardíacas e os óbitos maternos tiveram associação significativa (p < 0,05) com as variáveis OMSm, CF-NYHA, intervenções prévias à gestação, cardiopatias congênitas, cardiomiopatias, IC, disfunção de enxertos/próteses, fração de ejeção reduzida e HP, todas relacionadas a um maior risco materno ( Tabelas Suplementares 8A e 8B ). A subanálise comparativa entre as cardiomiopatias idiopática e periparto não mostrou diferenças quanto as complicações ou óbitos maternos ({9 (45%) versus 5 (37%)}: p = 0,43).

Os resultados obstétricos, relacionados às informações de 571 pacientes, mostraram que o parto cesárea predominou, com 548 (96,6%) recém-nascidos vivos, 146 (25,7%) prematuros e 39 (8,4%) com complicações no período neonatal, sendo que nove deles tiveram diagnóstico de cardiopatia congênita ( Tabela Suplementar 8 ).

A análise de predição de complicações cardíacas e óbitos maternos selecionou as variáveis significativas ( Tabelas Tabelas suplementares 8A e 8B ) para a construção dos modelos de predição ( Figuras 4A/B e Tabela 10A suplementares ) e indicou a OMSm como covariável independente de risco de complicações cardíacas e/ou óbitos ( Tabela Suplementar 10B ). A análise da predição de complicações obstétricas e fetais resultou no modelo final representado nas Figuras 5A/B e Tabela 11 Suplementares .

## Discussão

O REBECGA, primeiro registro nacional multicêntrico sobre doenças cardíacas na gestação, reúne dados de seis centros especializados no Brasil ( Figura Central ). Este estudo pioneiro trouxe uma visão abrangente da mortalidade materna associada às cardiopatias, incluindo óbitos tardios até 12 meses após a gestação, e dados valiosos sobre prevalência, complicações e fatores preditivos, permitindo comparações com registros internacionais, como o M-PAC^10^e o ROPAC^16^ e considerando particularidades culturais, sociais e demográficas brasileiras.

Inicialmente, a análise dos dados demográficos deste estudo mostrou a média de idade de 30,4 anos, refletindo o aumento da idade materna das gestantes brasileiras nesta última década^17^ e um percentual de 66,7% de mulheres brancas, superior aos 43,5% no Censo Nacional de 2022^18^ ( Tabela Suplementar 2 ).

### Prevalência e características das doenças cardíacas

As doenças valvares foram as mais comuns entre as lesões estruturais do coração, principalmente as da valva mitral. A etiologia reumática foi observada em 168 pacientes (69,7%), o que reflete a realidade brasileira^14^assemelhando-se ao registrado em outros países emergentes.^10 , 16^

O predomínio da doença reumática neste estudo pode justificar o elevado percentual de pacientes graves (46%), o que explica as taxas de complicações cardíacas (42,9%) e de óbitos maternos (3,9%). De fato, a história natural da doença valvar reumática favorece o desenvolvimento de complicações, como FA e HP, resultando em piores desfechos da gestação.^19^ É importante destacar que esses desfechos ocorreram mesmo em pacientes com CF I/II-NYHA, relatada por 83,7% delas no início da gestação ( Tabela Suplementar 7 ).

A escolha da PB em 74% das pacientes submetidas a implante valvar reflete o princípio amplamente adotado pela maioria dos serviços de cirurgia cardíaca no Brasil, conforme indicado nas diretrizes sobre doenças valvares.^20^ Essa abordagem favorece a PB em mulheres em idade reprodutiva, visto que essa opção não exige o uso contínuo de anticoagulantes. No entanto, do ponto de vista estrutural, o presente estudo mostrou a associação da disfunção da prótese com complicações e óbitos maternos, sugerindo que a PB não garante segurança à gestação ( Tabelas 6 e 8 B suplementares ).

A prevalência de cardiopatias congênitas foi de 35,7%, superior aos 19,1% relatados em um registro brasileiro da década de 1990^9^ mas ainda abaixo de outros registros internacionais.^16 , 21^ Ao incluir os 16,6% de doenças valvares congênitas, os dados se aproximaram dos padrões internacionais e superaram as cardiopatias reumáticas, antes predominantes no Brasil. Esses resultados refletem uma transição epidemiológica das doenças cardíacas em pacientes jovens no país, impulsionada pelo avanço no diagnóstico e tratamento de cardiopatias congênitas na infância. O predomínio de lesões congênitas com *shunts* em 33,8% dos casos está em concordância com a prevalência global das cardiopatias congênitas no adulto.^21^ Vale destacar o número crescente de gestantes com cardiopatias complexas, com bons resultados na gravidez.^22^

A mortalidade materna por cardiopatias congênitas é baixa, especialmente em países desenvolvidos.^23^ Neste estudo, essas cardiopatias apresentaram menor risco de complicações (OR 0,47; IC 95% 0,29-0,75; p = 0,002), possivelmente devido às correções cirúrgicas realizadas antes da gestação em 74,1% dos casos. Nesse cenário, merece destaque o óbito materno em paciente com comunicação interventricular não operada e HP grave (caso 17), evidenciando a importância da intervenção cirúrgica precoce nessa população.

As arritmias cardíacas sem lesão cardíaca estrutural, na maioria supraventriculares, tiveram bom controle medicamentoso ou por intervenções percutâneas, resultando em uma evolução favorável da gravidez. É notável que os avanços na eletrofisiologia, tanto no diagnóstico quanto no tratamento das arritmias, favoreçam um aumento significativo no percentual de gestantes com arritmias e bom prognóstico da gestação.^24^Como contrapartida, arritmias associadas à lesão cardíaca estrutural, foram, neste estudo, a segunda causa de complicações, atrás apenas da IC ( Tabela Suplementar 7 ). Em destaque, a FA, presente em 7,6% dos casos, esteve envolvida em dois dos 18 óbitos maternos.

No grupo das cardiomiopatias, observou-se um perfil desfavorável desde o início da gestação, evidenciado pela presença de fatores complicadores em 70,8% dos casos e por eventos cardíacos prévios em 50% das pacientes ( Tabela Suplementar 7 ). Essas condições contribuíram para os óbitos registrados em cinco casos, todos no pós-parto, devido à IC, resultando em uma taxa de mortalidade de 6,8%. De forma geral, as cardiomiopatias tiveram forte associação a complicações e óbitos maternos (OR 2,32; IC 95% 1,25-4,24; p = 0,007), independentemente da etiologia, reforçando a constatação de estudo anterior.^25^

Uma particularidade do REBECGA foi a inclusão de seis casos (0,94%) de cardiomiopatia chagásica, número muito inferior aos 128 casos (12,8%) registrados no Brasil na década de 1990, quando a doença de Chagas causou uma taxa de mortalidade de 8,3% entre gestantes com cardiomiopatias.^9^ No estudo atual, as formas arritmogênica e de IC da doença não se associaram aos óbitos maternos. Esses resultados refletem avanços no diagnóstico, no controle da transmissão e no tratamento dos pacientes.^26^ No entanto, estudos recentes sugerem possível subnotificação da doença, com um número maior de mulheres em idade reprodutiva infectadas, representando um risco significativo para mãe e feto.^27 , 28^

As doenças da aorta **,** apesar de sua baixa prevalência, apresentaram uma taxa de mortalidade materna de 14,2%, resultando em dois óbitos entre os 14 casos estudados. Esses dados ressaltam o alto risco dessas doenças para a gravidez.^29^ Além disso, as doenças da aorta geralmente ocorrem associadas a síndromes hereditárias, como a síndrome de Marfan, ou a doenças sistêmicas, como a arterite de Takayasu, a outras cardiopatias congênitas.^30^

A doença cardíaca isquêmica foi observada em gestantes com uma média de idade de 37,0 ± 7 anos, representando 3,6% das cardiopatias registradas. É importante mencionar que, entre as 23 pacientes, 65,2% apresentavam histórico de eventos cardíacos isquêmicos, enquanto as demais sofreram infarto durante ou após a gravidez.

Vale destacar que mais da metade dessas pacientes teve complicações obstétricas e/ou fetais, provavelmente devido à natureza do tratamento emergencial dessa condição.^31^ De fato, a cardiopatia isquêmica foi a variável mais significativa na predição de complicações obstétricas e fetais nas análises univariada (OR 5,61; IC 95%: 1,48-36,6; p = 0,026) e multivariada (OR 5,75; IC 95%: 1,24-26,7; p = 0,022).

### Considerações sobre as complicações cardíacas e óbitos maternos

O registro REBECGA revelou uma taxa de complicações maternas de 28%, abaixo dos 36% observados em outros países emergentes,^16^ mas próxima aos 23,5% de um estudo brasileiro de 1990.^9^ Isso sugere que, apesar das mudanças epidemiológicas, os desafios permanecem. A IC continua sendo a complicação mais frequente, com prevalência de 16,6%, refletindo tanto o impacto hemodinâmico da gravidez^32^ quanto o atraso no diagnóstico e nas intervenções.^33^

As evidências indicam que o risco de complicações e óbitos maternos aumenta proporcionalmente com a elevação da CR-OMSm, já validada como um bom preditor de risco.^12^ No entanto, este estudo revelou, de forma curiosa, uma taxa expressiva (26%) de complicações entre as pacientes classificadas como CR II-OMS, sugerindo a necessidade de estudos futuros para aprimorar a aplicação da CR-OMSm na estratificação de risco em cardiopatias.

O percentual de óbitos maternos foi de 2,8%, diferente do observado em outros estudos,^8 , 10 , 16^ possivelmente devido à inclusão de um período de 12 meses após o parto, enquanto os demais consideraram apenas o puerpério ou até 90 após a gestação. Dos 18 óbitos, 10 (55,5%) ocorreram no puerpério, e cinco (27,8%) foram classificados como mortalidade materna tardia. Isso destaca o pós-parto como um período crítico, evidenciando a necessidade de otimizar o tratamento cardiovascular e garantir cuidados especializados. Além disso, a infecção por COVID-19 precedeu a morte de quatro pacientes, corroborando os dados do Ministério da Saúde, que reportaram uma mortalidade de 13,5% entre gestantes e puérperas infectadas, das quais 25% eram portadoras de cardiopatias.^34^

Variáveis preditivas de complicações cardíacas e óbitos maternos

A análise de predição das complicações e óbitos maternos ressaltou variáveis que refletem o perfil clínico crítico das pacientes incluídas neste registro, muitas delas com contraindicação formal à gravidez ( Suplementar 10A ). Apesar de 62,5% das pacientes terem recebido orientação cardiológica prévia sobre os riscos associados à gestação, apenas 32,5% planejaram a gravidez, evidenciando uma lacuna significativa no planejamento reprodutivo dessas mulheres.

Entre as variáveis selecionadas, a HP merece destaque especial, pois manteve-se como um forte preditor de complicações e óbitos maternos ( Figura 4A e Tabela 10A suplementar ) corroborando a literatura internacional. Um estudo com 3.532 gestantes relatou taxas mais altas de eventos cardiovasculares (15,7% vs. 0,3%; p < 0,001) e mortalidade materna (0,9% vs. 0,01%; p < 0,001) em mulheres com HP.^35^ Esses dados reforçam que, apesar dos avanços terapêuticos e melhora do prognóstico, a gravidez continua sendo uma condição de alto risco para mulheres com HP, sendo fortemente desaconselhada nessa população.

### Medicamentos prescritos

A escolha das medicações utilizadas na gestação e no pós-parto ( Tabela Suplementar 5 ), no tratamento das complicações cardíacas maternas, fundamentou-se nas recomendações convencionais.^5 - 7 , 36^ Ressalta-se que os antagonistas dos receptores de angiotensina e os inibidores da enzima conversora de angiotensina, conhecidos por seus efeitos teratogênicos, não foram prescritos durante a gravidez. Contudo, nos casos de gravidez não planejada, as pacientes que já faziam uso desses medicamentos, o tratamento foi imediatamente suspenso na primeira consulta da gestação.

### Considerações sobre os resultados obstétricos e fetais

O parto cesárea foi realizado em 62,9% das pacientes, alinhando-se à realidade brasileira, onde, segundo dados do Ministério da Saúde, cerca de 57% dos partos são realizados por cesariana, o que equivale a cinco vezes as taxas recomendadas pela OMS, que são de 10% a 15%.^37^ Neste estudo, a escolha do tipo de parto foi baseada na indicação da equipe obstétrica, reconhecendo-se as indiscutíveis vantagens do parto vaginal, que incluem menores riscos de sangramento e infecção, além de uma recuperação mais rápida, resultando em menor ocorrência de complicações. A incidência de partos prematuros de 25%, superior aos 11% reportados na população geral brasileira,^38^ possivelmente relacionada às condições clínicas maternas e à evolução obstétrica, que muitas vezes demandam a antecipação do parto. A ocorrência das perdas fetais em 6,3% das pacientes não reflete plenamente a realidade das gestantes cardiopatas, já que, em estudos retrospectivos, informações sobre perdas fetais precoces podem ser subnotificadas. Por outro lado, o índice de hipertensão gestacional, observado em apenas 1,1%, foi significativamente menor do que o estimado para a população geral.^39^

### Limitações

As limitações do estudo refletem características inerentes a pesquisas retrospectivas, muitas vezes não permitindo embasar discussões aprofundadas sobre alguns tópicos. Nesse contexto, a supervisão do centro regulador da pesquisa (Academic Research Organization – ARO) desempenhou papel crucial na filtragem dos dados, uniformização dos diagnósticos, agrupamento das complicações e padronização dos tratamentos, garantindo a conclusão desta etapa com qualidade. Ressalta-se que a temporalidade dos dados esteve limitada ao período de 2017 a 2021 para todos os centros, restringindo a análise de tendências ao longo do tempo. O REBECGA foi idealizado para incluir hospitais de todas as macrorregiões do Brasil, com financiamento do Ministério da Saúde, empenhado em 2022, mas suspenso em 2023. Ainda assim, seis centros seguiram com a coleta e análise dos dados com recursos próprios, concluindo a etapa retrospectiva.

## Conclusões

O REBECGA evidenciou a alta prevalência da doença valvar durante a gestação, identificou a insuficiência cardíaca e as arritmias como as principais complicações cardíacas e revelou que a maioria das mortes maternas ocorreu no puerpério. Além disso, o estudo destacou que as condições classificadas como risco IV pela OMS, a CF III/IV da NYHA e a hipertensão pulmonar foram significativamente associadas à predição de complicações e óbitos maternos. O REBECGA representa um marco importante na pesquisa sobre cardiopatias e gravidez no Brasil, e sua continuidade fortalecerá as bases para estudos futuros, contribuindo para a redução da mortalidade materna associada a essas condições.
