# Supplementary material for: REBECGA: Registro Brasileiro de Cardiopatia e Gravidez. Estudo Multicêntrico Epidemiológico sobre Cardiopatias na Gravidez: Etapa Retrospectiva
Source: Arq Bras Cardiol. 2025 Aug 6;122(8):e20240807. [Article in Portuguese] doi: 10.36660/abc.20240807 (PMC12677860; doi:10.36660/abc.20240807)
Supplement: Translation

# Translation: REBECGA: Brazilian Registry of Heart Disease and Pregnancy. Multicenter Epidemiological Study of Heart Diseases in Pregnancy: Retrospective Cohort

**DOI:** 10.36660/abc.20240807i

**Authors:** Walkiria Samuel Avila, Alexandre Jorge Gomes de Lucena, Claudia Maria Vilas Freire, Fabio Bruno da Silva, Ivan Romero Rivera, Juliana Rodrigues Soares Oliveira, Maria Alayde Mendonça Rivera, Regina Coeli de Carvalho, Vicente Avila, Flávio Tarasoutchi

**Affiliations:** 1 Brazil Instituto do Coração do Hospital das Clínicas da Faculdade de Medicina da Universidade de São Paulo, São Paulo, SP – Brazil; 2 Brazil Hospital Agamenon Magalhães, Recife, PE – Brazil; 3 Brazil Universidade Federal de Minas Gerais, Belo Horizonte, MG – Brazil; 4 Brazil ECOCENTER, Belo Horizonte, MG – Brazil; 5 Brazil Instituto Dante Pazzanese de Cardiologia, São Paulo, SP – Brazil; 6 Brazil Universidade Federal de Alagoas Faculdade de Medicina, Maceió, AL – Brazil; 7 Brazil Secretaria de Estado da Saúde do Ceará (SESA), Fortaleza, CE – Brazil; 8 Brazil Hospital do Servidor Público Municipal, São Paulo, SP – Brazil

**Keywords:** Registry, Heart Diseases, Pregnancy, Maternal Mortality

## Abstract

**Background:**

Heart disease, which affects approximately 4% of pregnancies, is the leading non-obstetric cause of maternal death. It is estimated that 40% of these deaths could be prevented with a better understanding of pregnancy risks.

**Objectives:**

To develop the Brazilian Registry of Heart Diseases in Pregnancy (REBECGA) to study the prevalence, complications, and maternal mortality associated with heart diseases during pregnancy and postpartum.

**Methods:**

A multicenter retrospective study of pregnancy and postpartum follow up until 12 months after delivery in women with heart diseases, including analysis of predictive variables for maternal outcomes. A two-tailed significance level of 5% was adopted for statistical analysis.

**Results:**

A total of 638 pregnant women were included, with the following diagnoses: valvular disease (37.8%), congenital heart diseases (35.7%), arrhythmias without structural heart damage (14.7%), cardiomyopathies (11.3%), and other heart diseases (6.4%). The main complications were heart failure (16.7%), arrhythmia (10.7%), and syncope (8.0%), with 18 maternal deaths (2.8%), 10 of which occurred during the postpartum period. Cesarean delivery was indicated in 62.9% of cases, with 25.7% preterm births and 3.2% fetal losses. Multivariate analysis identified WHO class IV risk classification (OR 3.51; 95% CI 1.75–7.04; p < 0.001); NYHA class III/IV (OR 2.27; 95% CI 1.12–4.60; p = 0.023), and pulmonary hypertension (OR 2.45; 95% CI 1.05–5.70; p = 0.037) as predictive variables for maternal complications and deaths.

**Conclusions:**

REBECGA highlighted the prevalence of valvular disease, heart failure as the main complication, the postpartum period as a critical time for maternal deaths, and identified predictive risk variables for adverse pregnancy outcomes in women with heart disease.

## Introduction

Heart diseases affect about 4% of pregnancies in Brazil,^1^being the main non-obstetric cause of maternal death during pregnancy and after childbirth.^2^This scenario results from the increased prevalence of cardiovascular risk factors in young women, the postponement of pregnancy to older ages, and the greater survival of women with heart diseases manifested in childhood and adolescence.

About 80% of maternal deaths from heart diseases are preventable, with two-thirds occurring in the first 12 months postpartum.^3^These deaths are often attributed to the lack of early diagnosis and specialized treatment, and failures in the healthcare system.^4^

Most recommendations on heart disease and pregnancy are based on level C evidence, mainly grounded in expert opinions and multicenter records.^5 - 11^In this context, the modified World Health Organization (mWHO) classification has shown good sensitivity in predicting maternal complications, being a valuable tool for both pre-conception counseling and in managing pregnancies of women with heart disease^5 , 12^( Supplementary Table 1 ).

However, gaps persist in Brazil, where international scores are limited due to epidemiological, social, and demographic differences.^13^In this scenario, the creation of the Brazilian Registry of Heart Disease and Pregnancy (REBECGA, *Registro Brasileiro de Cardiopatia e Gravidez* ) emerges as a necessity to provide a realistic view of heart diseases in pregnancy in Brazil, aligning the country with global initiatives.

The main objective of REBECGA is to determine the prevalence of heart diseases, cardiac complications, and maternal mortality related to pregnancy. It also aims to identify predictive factors of complications and maternal deaths, as well as describe obstetric and fetal outcomes.

## Methods

REBECGA is a multicenter observational study accessible to hospitals throughout Brazil with adequate infrastructure to monitor women with a previous diagnosis of heart disease, from pregnancy to 12 months postpartum, even if they participate in other clinical trials. The 12-month postpartum was considered in order to identified the circumstances of late maternal mortality, conceptually defined as up to 12 months after the end of pregnancy. The study consists of two stages: 1) a retrospective stage, already completed, with data from pregnant women attended between 2017 and 2020 (content of this publication); 2) a prospective stage, ongoing, with completion expected in 2027.

The data considered for the study were: 1) diagnosis of heart disease; 2) mWHO risk classification (RC I, II, II-III, IV - mWHO); 3) New York Heart Association functional class (FC I, II, III, IV-NYHA); 4) complicating factors – ventricular dysfunction, pulmonary hypertension (PH), hypoxemia, atrial fibrillation (AF), residual lesions after surgical or percutaneous intervention, graft or prosthesis dysfunction; 5) cardiac events prior to pregnancy; 6) history of surgery or intervention; and 7) obstetric history.

The exclusion criteria for the study were: patients without a defined diagnosis or under investigation for heart disease, PH without primary cardiac involvement, and cardiac arrhythmias without documented records.

The analysis of disease progression during the trimesters of pregnancy, in the postpartum period (up to 42 days after childbirth), and up to 12 months after the end of pregnancy considered the occurrence of: 1) cardiac complications – heart failure (HF), “new” arrhythmia, thromboembolism, hypoxemia, syncope, acute myocardial infarction, aortic dissection, infectious endocarditis); 2) maternal deaths; 3) hospitalization for treatment of cardiac complications; 4) need for interventional treatment (surgical or percutaneous); and 5) obstetric complications grouped into miscarriage, gestational hypertension, postpartum hemorrhage, and infection. Maternal deaths were analyzed “on a case-by-case basis”, considering the clinical characteristics prior to pregnancy, cause of death, and fetal survival.

The adopted approaches, including general and preventive measures, pharmacological and/or interventional therapy, followed the conventional recommendations established by the Guidelines on Heart Disease and Pregnancy.^5 - 7^

A descriptive analysis was carried out on the information related to types of delivery, anesthesia used, and newborn data, including fetal losses, prematurity, and congenital heart diseases, based on information from patients’ self-reports or medical records.

Analyses were conducted to assess the relationship between heart diseases, patients’ baseline characteristics, complications, and maternal deaths.

### Data recording and management

The data were recorded on the Research Electronic Data Capture (REDCap) platform^14^by researchers from each participating center and monitored by the Academic Research Organization (ARO) of the Heart Institute (InCor) – FMUSP. The study was observational and descriptive, without interventions and without risks to the participants, and was approved by the Research Ethics Committee (CEP) of the General Hospital of the University of São Paulo Medical School (HCFMUSP) (protocol numbers SDC5232/21/007 CAAE: 42739321.9.1001.0068).

### Statistical analysis

Statistical analysis was performed using the R 4.2.2 software and graphs were created with the ggplot2 package.^15^Continuous variables were presented as means and standard deviations or as medians and interquartile ranges, depending on the distribution. Normality of the distribution was assessed using the Kolmogorov-Smirnov test. Categorical variables were described as absolute and relative frequencies. Fisher’s exact test and the chi-square test were used to evaluate associations between categorical variables. One-way ANOVA and unpaired t-tests or Wilcoxon-Mann-Whitney tests were used to compare continuous variables between the groups. A binary logistic regression model was adjusted to identify variables associated with the occurrence of complications and/or maternal death, with initial selection of variables with p < 0.10 in the univariate test and stepwise method. For the evaluation of the predictive performance of the models, ROC curves were considered. For statistical tests, a significance level of 5% was adopted and all were considered as two-tailed.

## Results ( Central Illustration )

Between 2017 and 2020, data from 638 pregnant women with heart disease were included in the retrospective phase of the REBECGA Registry, which involved six cardiology centers ( Central Illustration and Supplementary Figure 1 ). The study participants had an mean age of 30.4 ± 7.3 years, with a predominance of white and single women. Half had completed elementary or high school, and 62.7% reported previous pregnancies ( Supplementary Table 2 ). Regarding prior reproductive counseling, only 32.2% had planned the pregnancy, 57.3% had received guidance on contraceptive methods, and 64.9% had been advised about the risks of pregnancy.

The analysis of the distribution of heart diseases ( Supplementary Figure 2 ) highlighted the prevalence of valvular disease, which was very close to that of congenital heart disease, and a percentage of arrhythmias without structural lesions higher than that of cardiomyopathies. There was a predominance of rheumatic mitral valve disease, with previous surgical or percutaneous interventions before pregnancy most frequently involving the implantation of a biological prosthesis (BP) ( Supplementary Figure 3A ).

Among congenital heart diseases, combined lesions, intracavitary communications (“shunts”), and tetralogy of Fallot were predominant as cyanogenic heart diseases ( Supplementary Figure 3B ).

In the group of cardiomyopathies, the dilated phenotype was the most frequent and similarly distributed between idiopathic and peripartum ( Supplementary Figure 3C ). Among isolated arrhythmogenic diseases, supraventricular arrhythmias were the most common; 53 (56.4%) patients had undergone percutaneous intervention before pregnancy ( Supplementary Figure 3D ).

The clinical characteristics of the patients at the first pregnancy visit ( Supplementary Tables 3 A, B, C, D, E ) showed that about one-third (36.1%) of patients were classified as mWHO risk III/IV ( Central Illustration ); 71 out of 598 patients (11.2%) were in NYHA FC III-IV. A total of 224 complicating factors (36.5%) and 187 previous cardiac events (29.2%) were identified among the included patients, with some cases presenting more than one factor or event.

The longitudinal analysis of maternal course ( Supplementary Table 4 ) over the pregnancy trimesters showed the evolution of NYHA FC up to the postpartum period, with the need for hospitalizations, therapeutic adjustments, and indications for surgical or percutaneous interventions in cases refractory to clinical treatment. The most frequent cardiac complications were HF, arrhythmias, and syncope. Over the 12 months following pregnancy, the complication rate remained high, and more than half of maternal deaths occurred in the postpartum period, with a total of 10 out of 18 (55.6%) fatal occurrences. The frequency of medications prescribed during pregnancy and after childbirth, based on the analysis of 602 patients, is summarized in Supplementary Table 5 .

Eighteen (2.8%) maternal deaths were recorded, three (16.6%) during pregnancy, 10 in the postpartum period (55.5%), and five (27.7%) over the 12 months following the postpartum period, described case by case ( Supplementary Table 6 ). Cases 8, 9, 10, and 14 had COVID-19 infection preceding maternal death, and one stillbirth (case 10).

Three emergency procedures were performed: 1^st^) case 13 – correction of aortic dissection with coronary implantation at the 36th week of pregnancy after the delivery of a live and healthy fetus, resulting in maternal death on the second postoperative day; 2^nd^) case 10 – reoperation for mitral valve replacement due to prosthesis calcification on the 30th postpartum day, resulting in death on the second postoperative day; 3^rd^) case 12 – percutaneous “valve in valve” procedure at the 12th week of pregnancy in a patient with a calcified prosthesis and acute HF, resulting in death on the first postoperative day.

The analysis of the baseline characteristics of the patients and the frequency of maternal and fetal complications showed significant differences (p < 0.05) between the types of heart disease for the variables age, mWHO, NYHA FC, complicating factors, history of cardiac events, and interventions prior to pregnancy ( Supplementary Table 7 ).

Cardiac complications and maternal deaths were significantly associated (p < 0.05) with the variables mWHO classification, NYHA FC, interventions prior to pregnancy, congenital heart diseases, cardiomyopathies, HF, graft/prosthesis dysfunction, reduced ejection fraction, and PH, all related to higher maternal risk ( Supplementary Tables 8A and 8B ). The comparative sub-analysis between idiopathic and peripartum cardiomyopathies showed no significant differences in complications or maternal deaths ({9 [45%] vs. 5 [37%]}: p = 0.43).

The obstetric outcomes related to information from 571 patients showed that cesarean delivery was the most frequently performed, with 548 (96.6%) live births, 146 (25.7%) premature, and 39 (8.4%) presenting complications in the neonatal period, nine of whom were diagnosed with congenital heart disease ( Supplementary Table 8 ).

The analysis of the predictors of cardiac complications and maternal deaths selected significant variables ( Supplementary Tables 8A and 8B ) for the construction of prediction models ( Supplementary Figures 4A/B and Table 10A ) and indicated mWHO as an independent covariate of risk for cardiac complications and/or deaths ( Supplementary Table 10B ). The analysis of the prediction of obstetric and fetal complications resulted in the final model presented in Supplementary Figures 5A/B and Table 11 .

## Discussion

The REBECGA, the first national multicenter registry on heart diseases during pregnancy, gathers data from six specialized centers in Brazil ( Central Illustration ). This pioneering study provides a comprehensive view of maternal mortality associated with heart disease, including late deaths up to 12 months after pregnancy, and as well as on prevalence, complications, and predictive factors, allowing comparisons with international registries such as M-PAC^10^and ROPAC,^16^considering Brazilian cultural, social, and demographic particularities.

Initially, the analysis of demographic data from this study showed an average age of 30.4 years, reflecting the increase in maternal age of Brazilian pregnant women in the last decade,^17^and a proportion of 66.7% of white women, higher than the 43.5% in the National Census of 2022^18^(Supplementary Table 2).

### Prevalence and characteristics of heart diseases

Valvular diseases were the most common conditions among structural heart lesions, mostly involving the mitral valve. Rheumatic etiology was observed in 168 patients (69.7%), reflecting the Brazilian reality^14^and resembles records from other emerging countries.^10 , 16^

The predominance of rheumatic disease in this study may explain the high percentage (46%) of severe patients, which in turn explains the rates of cardiac complications (42.9%) and maternal deaths (3.9%). Indeed, the natural history of rheumatic valvular disease favors the development of complications, such as AF and PH, resulting in worse pregnancy outcomes.^19^It is important to note that these outcomes occurred even in NYHA FC, I/II reported by 83.7% of patients at the beginning of pregnancy (Supplementary Table 7).

The choice of BP in 74% of patients undergoing valve implantation reflects the principle widely adopted by most cardiac surgery services in Brazil, as indicated in the guidelines on valvular diseases.^20^This approach favors BP in women of reproductive age, as this option does not require continuous use of anticoagulants. However, from a structural point of view, the present study showed the association between prosthesis dysfunction and complications and maternal deaths, suggesting that BP does not guarantee safety during pregnancy (Supplementary Tables 6 and 8B).

The prevalence of congenital heart disease was 35.7%, higher than the 19.1% reported in a Brazilian registry from the 1990s,^9^but still below other international registries.^16 , 21^Including the 16.6% of congenital valvular diseases, the data aligned more closely with international standards and surpassed rheumatic heart diseases, previously predominant in Brazil. These results reflect an epidemiological transition of heart diseases in young patients in the country, driven by advances in the diagnosis and treatment of congenital heart diseases in childhood. The predominance of congenital lesions with shunts in 33.8% of cases is in line with the global prevalence of congenital heart diseases in adults.^21^It is worth noting the increasing number of pregnant women with complex heart diseases, with good pregnancy outcomes.^22^

Maternal mortality due to congenital heart diseases is low, especially in developed countries.^23^In this study, these heart diseases presented a lower risk of complications (OR 0.47; 95% CI 0.29–0.75; p=0.002), possibly due to surgical corrections performed before pregnancy in 74.1% of cases. In this context, the maternal death of a patient with unoperated ventricular septal defect and severe PH (case 17) highlights the importance of early surgical intervention in this population.

Cardiac arrhythmias without structural heart lesions, mostly supraventricular, were well controlled through medication or percutaneous interventions, resulting in a favorable pregnancy outcome. It is notable that advances in electrophysiology, both in the diagnosis and treatment of arrhythmias, have contributed to in the percentage of pregnant women with arrhythmias and a pregnant women with arrhythmias and a good pregnancy prognosis.^24^Conversely, arrhythmias associated with structural heart lesions were the second leading cause of complications in this study, behind only HF (Supplementary Table 7). Atrial fibrillation, present in 7.6% of cases, was involved in two of the 18 maternal deaths.

In the group of cardiomyopathies, an unfavorable profile was observed from the beginning of pregnancy, as evidenced by the presence of complicating factors in 70.8% of cases and previous cardiac events in 50% of patients (Supplementary Table 7). These conditions contributed to the five recorded maternal deaths, all in the postpartum period, due to HF, resulting in a mortality rate of 6.8%. Overall, cardiomyopathies were strongly associated with complications and maternal deaths (OR 2.32; 95% CI 1.25–4.24; p = 0.007), regardless of etiology, reinforcing the findings of a previous study.^25^

A particular feature of REBECGA was the inclusion of six cases (0.94%) of Chagas cardiomyopathy, a much lower number than the 128 cases (12.8%) reported in Brazil in the 1990s, when Chagas disease caused a mortality rate of 8.3% among pregnant women with cardiomyopathies.^9^In the current study, the arrhythmogenic and HF forms of the disease were not associated with maternal deaths. These results reflect advances in diagnosis, transmission control, and patient treatment.^26^However, recent studies suggest possible underreporting of the disease, with a higher number of women of reproductive age infected, representing a significant risk for both mother and fetus.^27 , 28^

Aortic diseases, despite their low prevalence, had a maternal mortality rate of 14.2%, resulting in two deaths among the 14 cases studied. These data highlight the high risk of these diseases for pregnancy.^29^Additionally, aortic diseases generally occur in association with hereditary syndromes, such as the Marfan syndrome, or systemic diseases, such as Takayasu arteritis, and other congenital heart diseases.^30^

Ischemic heart disease was observed in pregnant women with a mean age of 37.0 ± 7 years, representing 3.6% of registered heart diseases. It is important to note that, among the 23 patients, 65.2% had a history of ischemic cardiac events, while the remaining patients suffered a heart attack during or after pregnancy.

It is worth highlighting that more than half of these patients experienced obstetric and/or fetal complications, likely due to the emergency nature of the treatment for this condition.^31^In fact, ischemic heart disease was the most significant variable in predicting obstetric and fetal complications in univariate (OR 5.61; 95% CI: 1.48-36.6; p = 0.026) and multivariate analyses (OR 5.75; 95% CI: 1.24-26.7; p = 0.022).

### Considerations regarding cardiac complications and maternal deaths

The REBECGA registry reported a maternal complication rate of 28%, lower than the 36% observed in other emerging countries,^16^but close to the 23.5% reported in a Brazilian study conducted in 1990.^9^This suggests that, despite epidemiological changes, significant challenges remain. HF continues to be the most frequent complication, with a prevalence of 16.6%, reflecting both the hemodynamic impact of pregnancy^32^and delays in diagnosis and interventions.^33^

Evidence indicates that the risk of complications and maternal deaths increases proportionally with higher mWHO classifications, which have already been validated as a good risk predictor.^12^However, interestingly, this study revealed a significant rate (26%) of complications among patients classified as mWHO risk II, suggesting the need for future studies to improve the application of mWHO in risk stratification for heart disease.

The maternal mortality rate was 2.8%, differing from other studies,^8 , 10 , 16^possibly due to the inclusion of a 12-month postpartum period, whereas others considered only the puerperium or up to 90 days after pregnancy. Among the 18 deaths, 10 (55.5%) occurred during the puerperium, and five (27.8%) were classified as cases of late maternal mortality. This highlights the postpartum period as a critical phase, underscoring the need to optimize cardiovascular treatment and ensure access to specialized care. Moreover, COVID-19 infection preceded the death of four patients, corroborating data from the Brazilian Ministry of Health reported a 13.5% mortality rate among pregnant and postpartum women infected, 25% of whom had heart conditions.^34^

### Predictive variables of cardiac complications and maternal deaths

The analysis of predictors for complications and maternal deaths highlighted variables that reflect the critical profile of patients included in this registry, many of whom had formal contraindications to pregnancy (Supplementary Table 10A). Despite 62.5% of patients having received prior cardiology counseling on the risks associated with pregnancy, only 32.5% had planned their pregnancy, revealing a significant gap in reproductive planning among these women.

Among the selected variables, PH deserves special attention, as it remained a strong predictor of complications and maternal deaths ( Figure 4A and Supplementary Table 10A ), consistent with international literature. A study involving 3,532 pregnant women reported higher rates of cardiovascular events (15.7% vs. 0.3%; p < 0.001) and maternal mortality (0.9% vs. 0.01%; p < 0.001) in women with PH.^35^These findings reinforce that, despite therapeutic advances and improved prognosis, pregnancy continues to be a high-risk condition for women with PH and is strongly discouraged in this population.

### Prescribed medications

The choice of medications used during pregnancy and postpartum ( Supplementary Table 5 ) in the treatment of maternal cardiac complications was based on conventional recommendations.^5 - 7 , 36^Notably, that angiotensin receptor blockers and angiotensin-converting enzyme inhibitors (ACE inhibitors), known for their teratogenic effects, were not prescribed during pregnancy. However, in cases of unplanned pregnancy, the treatment with these medications was immediately discontinued at the first prenatal consultation.

### Considerations regarding obstetric and fetal outcomes

Cesarean delivery was performed in 62.9% of the patients, consistent with the Brazilian context, where, according to Ministry of Health data, approximately 57% of deliveries are cesarean sections, equivalent to five times the rates recommended by the WHO, which range from 10 to 15%.^37^In this study, the choice of delivery method was based on the recommendation of the obstetric team, recognizing the well-established advantages of vaginal delivery, which include lower risks of bleeding and infection, as well as faster recovery, resulting in fewer complications. The incidence of preterm deliveries was 25%, higher than the 11% reported in the general Brazilian population,^38^possibly related to maternal clinical conditions and obstetric evolution, often requiring early delivery. The occurrence of fetal losses in 6.3% of the patients does not fully reflect the reality of pregnant women with heart conditions, as early fetal losses may be underreported in retrospective studies. On the other hand, the rate of gestational hypertension observed at only 1.1% was significantly lower than that estimated for the general population.^39^

### Study limitations

The limitations of the study reflect typical constraints of retrospective designs, often not allowing for in-depth discussions on some topics. In this context, the supervision of the research regulatory center (Academic Research Organization – ARO) played a crucial role in data filtering, diagnostic standardization, complication grouping, and treatment harmonization, ensuring high-quality completion of this phase. It is important to note that the data collection period was limited to the period from 2017 to 2021 for all centers, restricting the analysis of trends over time. REBECGA was designed to include hospitals from all macro-regions of Brazil, with funding from the Ministry of Health allocated in 2022 but suspended in 2023. Nevertheless, six centers continued data collection and analysis using their own resources, completing the retrospective phase.

## Conclusions

REBECGA highlighted the high prevalence of valvular disease during pregnancy, identified HF and arrhythmias as the main cardiac complications, and revealed that most maternal deaths occurred during the postpartum period. Moreover, the study emphasized that conditions classified

as WHO risk class IV, NYHA functional class III/IV, and pulmonary hypertension were significantly associated with the prediction of maternal complications and deaths. REBECGA represents an important milestone in research on heart diseases and pregnancy in Brazil. Its continuity will strengthen the foundations for future studies, contributing to reducing maternal mortality associated with these conditions.
